# Nomos: An Attempt to Demonstrate a Central Physical Law in Nature

**Published:** 1856-10-01

**Authors:** 


### Part Third.

### Reviews.

*Nomos: an Attempt to demonstrate a Central Physical Law in Nature.* (Anonymous.) London: Longman and Co. 1856.

THIS is a very remarkable and clever book, equal in interest, but very different in its principles and objects, to the celebrated "Vestiges of the Creation." The author's speculations in the world of physical science will undoubtedly excite much attention among reading and philosophic men. He is undoubtedly an original thinker, and writes with great vigour and clearness. We are much mistaken if this volume has not a very large circulation. The author's object is to establish that the world of inorganic nature is ruled by one physical law, and not by several physical laws. He endeavours to demonstrate that the phenomena of electricity, magnetism, light, heat, chemical action and motion, are not to be understood unless they are regarded as signs of one and the same action, in ordinary matter. It is impossible, however, to give any accurate idea of the writer's views without going more into detail. The whole of the work must be read in order to be fully understood.

